# Management of non-specific thoracic spine pain: a cross-sectional study among physiotherapists

**DOI:** 10.1186/s12891-023-06505-8

**Published:** 2023-05-19

**Authors:** Marco Risetti, Riccardo Gambugini, Marco Testa, Simone Battista

**Affiliations:** Department of Neurosciences, Rehabilitation, Ophthalmology, Genetics, Maternal and Child Health, University of Genova, Campus of Savona, Via Magliotto 2, Savona, 17100 SV Italy

**Keywords:** Physical therapy modalities, Physical Therapy Specialty, Musculoskeletal Pain, Practice guidelines as topic, Education, Public Health Professional

## Abstract

**Background:**

The thoracic area has mainly been neglected in research compared to the lumbar and cervical regions. No clinical practice guidelines (CPGs) for non-specific thoracic spine pain (TSP) have been compiled. Therefore, it can be argued that the absence of specific CPGs raises questions about the management of non-specific TSP. Hence, this study aimed at determining the management of non-specific TSP among physiotherapists in Italy.

**Methods:**

A web cross-sectional survey investigating physiotherapists’ management of non-specific TSP was conducted. The survey instrument was divided into three sections. The first section obtained participants’ characteristics. The second section determined participants’ agreement with 29 statements regarding the clinical management of non-specific TSP utilising a five-point Likert scale. Participants who partially or completely agreed (scores 4–5) were considered to agree with the statements. A ≥ 70% of agreement with a statement was considered as consensus according to previous literature. The third section asked the participants to indicate how often they adopted several treatments to manage non-specific TSP with a 5-point scale (always – often – sometimes – rarely - never). The frequencies of answers were calculated, and a visual representation through a bar chart was reported. The online version of the survey instrument was delivered through the newsletter of the Italian Association of Physiotherapists and the postgraduate master’s degree in Rheumatic and Musculoskeletal Rehabilitation of the University of Genova (Genova, Italy).

**Results:**

In total, 424 physiotherapists (mean age (SD): 35.1 years (10.5); 50% women) completed the survey. In the second section, physiotherapists achieved consensus for 22/29 statements. Those statements addressed the importance of psychosocial factors, exercise, education, and manual therapy techniques in managing non-specific TSP. In the third section, 79.7% of participants indicated they would always adopt a multimodal treatment (education, therapeutic exercise, manual therapy), followed by education and information (72.9%), therapeutic exercise (62.0%), soft tissue manual therapy (27.1%), and manual therapy (16.5%).

**Conclusions:**

Study participants considered fundamentally using a multimodal programme based on education, exercise and manual therapy to manage non-specific TSP. This approach aligns with the CPGs for other chronic musculoskeletal pain than non-specific TSP.

**Supplementary Information:**

The online version contains supplementary material available at 10.1186/s12891-023-06505-8.

## Introduction

The thoracic spine has received less attention in terms of terminology, epidemiologic data and clinical management than the lumbar and cervical spines [1, 2]. As a result, Heneghan et al. referred to the thoracic spine as the “Cinderella” region of the spine [3]. As per the terminology, a shared definition of the non-specificity origin of thoracic spine pain (TSP) has yet to be made [4, 5]. Briggs et al. defined non-specific TSP as pain experienced in the region of the thoracic spine, between the thoracic levels T1–T12 and across the posterior side of the trunk [4]. Conversely, the International Association for the Study of Pain (IASP) refers to non-specific TSP as pain in the most lateral margins of the erector spinae muscles [5].

When it comes to the epidemiology of non-specific TSP, this condition is not well documented compared to neck pain (NP) and low back pain (LBP) [6, 7]. This phenomenon can be attributed to non-specific TSP being less considered as TSP is often a consequence of systemic and more severe conditions (e.g., cancers, fractures and infections) than musculoskeletal pain [8]. In fact, less than 35% of TSP has a musculoskeletal origin. As far as the management is concerned, no specific clinical practice guidelines (CPGs) for non-specific TSP were retrieved. Some recommendations made by Floren included education, exercise, passive physical modalities or multimodal interventions, as is the case for NP and LBP [9]. Several authors investigated the effectiveness of manual therapy in the short and long run, but no significant conclusions could be made due to the low quality of these studies [10–15].

Hence, it can be argued that the absence of specific CPGs raises questions about the management of non-specific TSP. To date, there is only one cross-sectional online survey study whose primary aim was to determine how physiotherapists manage TSP (either specific or non-specific) [16]. Without specific CPGs, physiotherapists might resort to managing TSP according to their preferences and beliefs or utilising LBP and NP CPGs that do not consider the uniqueness of TSP. Therefore, this study aimed at determining the management of non-specific TSP among physiotherapists in Italy.

## Methods

### Design

A web cross-sectional survey was developed according to the ‘International Handbook of Survey Methodology’ [17] to explore the management of non-specific TSP among physiotherapists in Italy. Specifically, the four authors (MR, RG, MT and SB) compiled the survey instrument. They are all physiotherapists who specialised in rheumatic and musculoskeletal disease (RMD) rehabilitation. SB is a joint PhD candidate in Neurosciences and Medical Science with proficiency in conducting web-based survey studies. MT has a PhD in Rehabilitation Science and Physiotherapy. MR compiled the first version of the survey instrument by starting from a previous survey-based study conducted in the United Kingdom [16], implementing and improving it with other questions adapted from LBP [18] and NP [19] CPGs as no non-specific TSP CPGs were found. To create the questions, information was retrieved on the different parts of clinical management (from the assessment to the treatment) of LBP and NP, after which a preliminary version composed of twenty-two questions were drafted by MR. RG, SB and MT scrutinised this version, and a few questions were simplified and deleted, reaching a consensus among the authors. After three rounds of revision, the final draft was compiled.

The survey instrument consisted of three sections and twenty questions (See Supplementary File 1). Section 1 – *Demographic, work, and academic characteristics of the sample* (questions 1 to 11): this section described the participants’ age, the gender they identified with, number of years as physiotherapists, highest educational attainments, predominant physiotherapy practice specialities (e.g., RMD, neurological etc.), health sector they were mostly working in (e.g., public, private etc.) and the mean number of people with non-specific TSP treated per year). Section 2 – *Agreement with the Statements regarding the Definition, Assessment, Importance of Psychological factors, and Treatment of Non-Specific TSP* (questions 12–19 ): this section comprised 29 statements about the definition of non-specific TSP (questions 12 and 13; statements 1–7), non-specific TSP assessment (questions 14 and 15; statements 8–17), relationship of non-specific TSP with psychosocial factors (e.g., kinesiophobia, pain catastrophising, mental health issues) (questions 16 and 17; statements 18–23) and non-specific TSP treatment (questions 18 and 19; statements 24–29) (Table 1). Participants indicated to what extent they agreed with the 29 statements with a 5-point Likert Scale. Section 3 – *Frequency of use of the treatments* (question 20). This section investigated how frequently the participants adopt several treatments to manage non-specific TSP. The interventions were categorised into manual therapy techniques (mobilisation, thrust), soft tissue manual therapy (massage, trigger point (TrP), pressure release, Muscle Energy Technique, Strain Counterstrain, Specific Soft Tissue Mobilisation), therapeutic exercises, patient education, and the use of a multimodal treatment thereof (manual therapy, therapeutic exercises, education).

**Table 1:** Section 2: Synopsis of the Statements Reported in the Survey Instrument (Sect. 2)

Statements	Bibliography
1) Non-specific TSP is experienced between the thoracic levels T1-T12 and the most lateral margins of the erector spinae muscles	IASP [5]
2) Non-specific TSP is experienced between the thoracic levels C7-T1 and T12-L1, centrally to the spine	Fouquet et al. [48]
3) Non-specific TSP is experienced in the region of the thoracic spine, between the thoracic levels T1–T12 and across the posterior side of the trunk	Briggs et al. [4]
4) It is not possible to identify clinically, via palpation and / or through instrumental tests a specific musculoskeletal structure as the source of pain	Wood et al. [49]
5) Non-specific TSP is experienced in thoracic region but has multisystemic origin	H.J. Myburgh [34]
6) It is not necessary to identify a specific musculoskeletal structure that can be defined as the source of non-specific TSP	Rock, J. M. & Rainey, C. E [50]
7) It is possible to identify clinically, via palpation and / or through instrumental tests a specific musculoskeletal structure that can be defined as the source of pain	H.J. Myburgh [34]
8) Chest imaging (MRI, CT, RX) is necessary to express the clinical diagnosis of non-specific TSP	Chou et al [51]; Blanpied, P. et al [52]; M. Nordin et al. [53]
9) Clinical interview is necessary to diagnose non-specific TSP clinically	Heneghan [16]
10) Manual tests are necessary to diagnose non-specific TSP clinically	Heneghan [16]
11) Physical examination is necessary to diagnose non-specific TSP clinically	Heneghan [16]
12) Active movements observation is necessary to diagnose non-specific TSP clinically	Heneghan [16]
13) Neurological examination is necessary to diagnose non-specific TSP clinically	Heneghan [16]; Skillgate et al. [12]
14) Passive range-of-motion examination is necessary to diagnose non-specific TSP clinically	Heneghan [16]
15) The soft tissue palpation is necessary to diagnose non-specific TSP clinically	Heneghan [16]
16) Regional and segmental joint provocation tests are necessary to diagnose non-specific TSP clinically	Heneghan [16]
17) Regional and segmental passive tests are necessary to diagnose non-specific TSP clinically	Heneghan [16]
18) Psychosocial factors should be investigated when it comes to non-specific musculoskeletal pain	Williams D.A [54]; Linton, S. J. et al. [55]
19) Kinesiophobia could influence non-specific TSP perception	Williams D.A [54]; Linton, S. J. et al. [55]
20) Pain catastrophising could influence the non-specific TSP perception	Williams D.A [54]; Linton, S. J. et al. [55]
21) Psychiatric disorders (e.g., anxiety disorders and major depression) could influence the non-specific TSP perception.	Williams D.A [54]; Linton, S. J. et al. [55]
22) Contextual and social factors could influence non-specific TSP perception	Williams D.A [54]; Linton, S. J. et al. [55]
23) Social factors work-related could influence non-specific TSP perception	Williams D.A [54]; Linton, S. J. et al. [55]
24) Generic exercise (i.e., generic physical activity, muscle stretching, strength/proprioceptive/muscular endurance exercises) is important in the short-term treatment	Heneghan [16]; Skillgate et al. [12]
25) Education and information are effective in the short-term treatment	Moseley et al [56]; Louw et al [57]; Nijs et al. [58]
26) Manual therapy (such as spinal manipulation, spinal and soft tissue mobilisation) is important in the short-term treatment	Schiller [11]; Skillgate [12]; Lehtola [13]; Pecos-Martin [14]; Heneghan et al. [16]
27) Generic exercise (i.e., generic physical activity, muscle stretching, strength/proprioceptive/muscular endurance exercises) is important in the long-term treatment	Heneghan [16]; Skillgate et al. [12]
28) Education and information are effective in the long-term treatment	Moseley et al. [56]; Louw et al. [57]; Nijs et al. [58]
29) Manual therapy (such as spinal manipulation, spinal and soft tissue mobilisation) is important in the long-term treatment	Schiller [11]; Skillgate [12]; Lehtola [13]; Pecos-Martin [14]; Heneghan et al. [16]

Legend: TSP, thoracic spine pain

Before the online dissemination, we further tested the survey instrument with two physiotherapists specialised in RMD rehabilitation to ensure the questions were understandable, relevant and comprehensive of the essential management of non-specific TSP. As no queries were raised and no changes were made to the survey instrument, further pilot testing was not performed. The online version of the survey instrument was delivered in Italian through Microsoft 365 Forms, a secure web application for creating online survey instruments, respecting the European General Data Protection Regulations (Supplementary File 1 – English Translation of the Survey Instrument) [20].

### Participants

The online version of the survey instrument was delivered through the Italian Association of Physiotherapists (AIFI) newsletters, its Manual Therapy Group division and the newsletters of the postgraduate master’s degree in RMD rehabilitation of the University of Genova (Genova, Italy). These groups were the target of recruitment since they were most likely to comprise physiotherapists who regularly treat RMD, including non-specific TSP. Additionally, participants were recruited via social media outlets. The survey instrument was available from 1 March to 31 August 2021. To be eligible, participants had to be physiotherapists enrolled in the Italian physiotherapist national register.

### Analysis

#### Section 1 – demographic, work, and academic characteristics of the sample

Demographic, work, and academic characteristics were analysed through descriptive analysis. Continuous variables (age) were reported as mean ± standard deviation (SD). Categorical variables (gender they identified with, number of years as a physiotherapist, the highest education attainment, predominant physiotherapy practice specialities, health sector they mainly worked in and mean number of people with non-specific TSP treated per year) were reported as absolute and percentage frequencies.

#### Section 2 - Agreement with the Statements regarding the Definition, Assessment, Importance of Psychological factors, and Treatment of Non-Specific TSP

To measure agreement with the abovementioned statements, a 5-point Likert scale ranging from completely disagree (score 1) to completely agree (score 5) was used [21]. Participants who partially or completely agreed (scores 4–5) were considered to agree with the statements. A ≥ 70% of agreement with a statement was considered as consensus according to previous literature [22–28]. The frequencies of the answers were calculated, and a visual representation through a bar chart was reported.

#### Section 3 – frequency of use of treatment techniques

Participants responded using a 5-point scale (always – often – sometimes – rarely - never) to indicate how frequently they would include the different interventions in managing non-specific TSP. The frequencies of answers were calculated, and a visual representation through a bar graph was reported.

### Sample size calculation

The sample size calculated for the study followed the calculation formula reported by Taherdoost et al. [29], and 370 participants were deemed necessary for an online survey with a confidence interval of 95% .

### Ethical and reporting considerations

The study was conducted following the Declaration of Helsinki principles. The Ethical approval for this study was obtained from the Ethics Committee for University Research (CERA: Comitato Etico per la Ricerca di Ateneo), University of Genova (approval date: 17/01/21; CERA2021.34). At the beginning of the survey instrument, participants’ consent to participate was gained after a brief cover letter, and the informed consent outlining the aim and duration of the study. Completion of the survey instrument was anonymous and entirely voluntary. IP addresses were not saved to ensure participant anonymity. Researchers’ contact details were supplied to enable any questions or concerns to be answered prior to completing the online survey instrument. Participants who did not consent to participate in the study were shown a ‘Thank-You page’ and could not continue. The study was reported following the ‘Strengthening the Reporting of Observational Studies in Epidemiology’ (STROBE) recommendations [30]. All the authors are responsible for the data acquisition and analysis, with the latter led by MR and SB.

## Results

A total of 427 physiotherapists accepted the invitation to participate in the study, and 424 participants completed the survey instrument. Three participants did not agree to provide informed consent and could not partake in the study. Demographic data are presented in Table 2.

**Table 2:** Participants’ demographic characteristics

Demographic Characteristics	
**Age** (years)(mean,(SD))	35.1 (10.5)
**Gender** (women); (men); (other) (N (%)):	212 (50); 211 (49,8); 1 (0.2)
**Years of Practice** (N (%)):	
Less than 1 year From 1 to 5 years From 6 to 10 years From 11 to 20 years More than 20 years	14 (3.3) 136 (32.1) 109 (25.7) 101 (23.8) 64 (15.1)
**Highest Educational Attainment** (N (%)):	
Postgraduate Master Degree* Master of Science (MSc)/Postgraduate II Level Degree† Doctor of Philosophy (PhD) Other Nothing	227 (53.5) 30 (7.1) 3 (0.7) 58 (13.7) 106 (25)
**Predominant physiotherapy practice specialties** (N (%)):	
Rheumatic and Musculoskeletal Diseases/orthopaedics	
Neurology Cardiorespiratory Geriatric Paediatric Pelvic floor Sport/Performing Arts Hand rehabilitation Management Research	316 (74.5) 33 (7.8) 7 (1.7) 42 (10) 9 (2.1) 5 (1.2) 5 (1.2) 3 (0.7) 1 (0.2) 3 (0.6)
**Health sector** (N (%)):	
Employee in public sector Employee in private sector Freelancer Coordinator Hospital Manager	65 (15.3) 71 (16.7) 294 (69.3) 4 (0.9) 13 (3.1) 3 (0.7)
**N° of people with non-specific TSP treated per year**:	
< 10 10–20 > 20	221 (52.1) 131 (30.9) 72 (17)72 (17)

Legend: N, number; %, percentage; *Academic degree that can be gained after BSc (Italian education system); † Academic degree that can be gained after MSc (Italian education system)

**Fig. 1:**
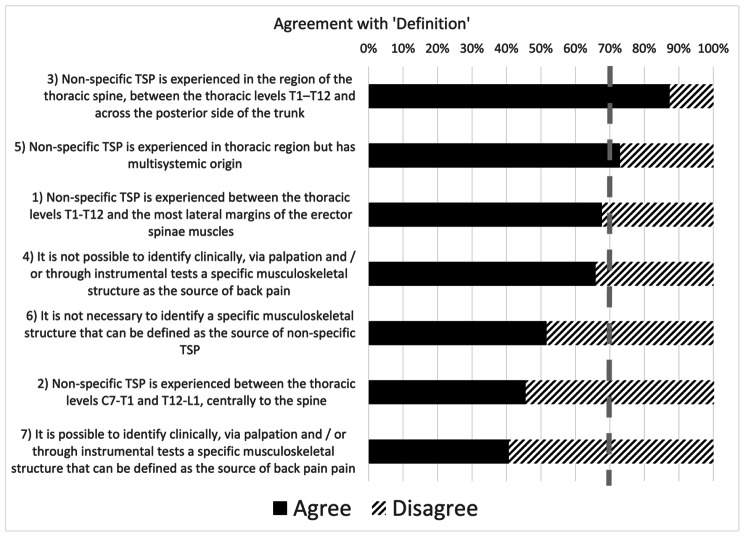
Levels of agreement among physiotherapists on the definition of non-specific thoracic spine pain. *The dashed grey line represents the consensus threshold set at 70%

**Fig. 2:**
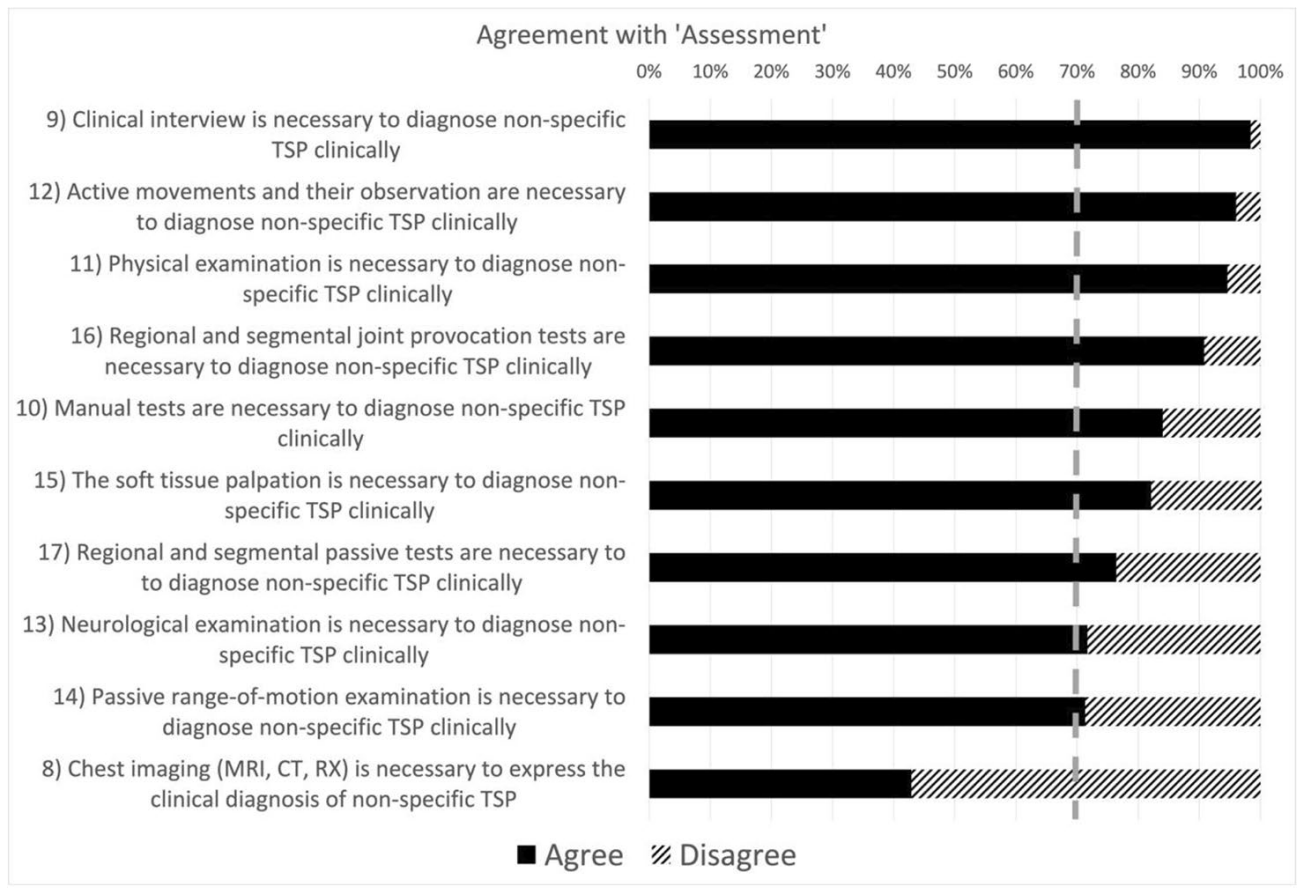
Levels of agreement among physiotherapists on the assessment of non-specific thoracic spine pain. *The dashed grey line represents the consensus threshold set at 70%

**Fig. 3:**
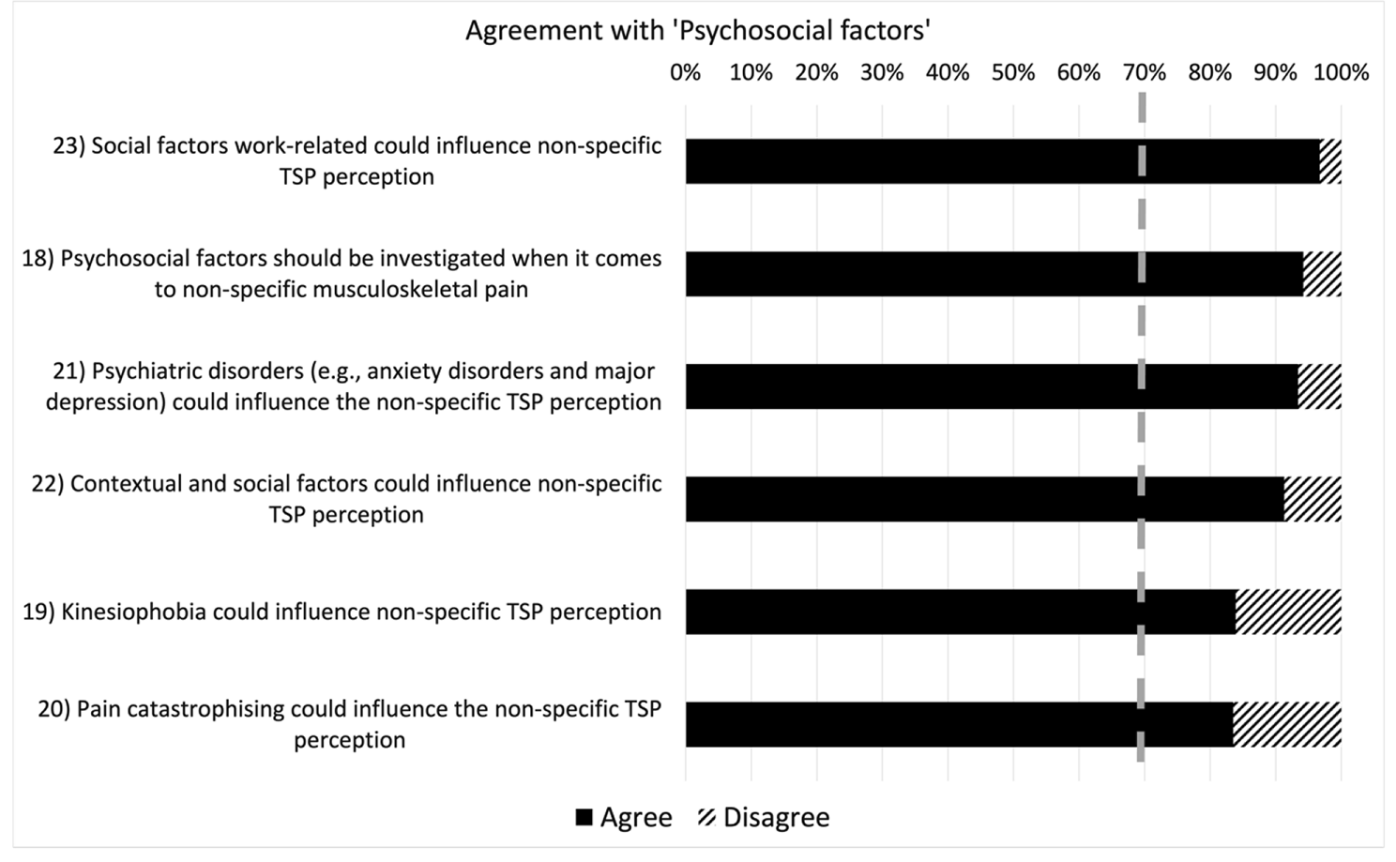
Levels of agreement among physiotherapists on the influence of psychosocial factors on non-specific thoracic spine pain. *The dashed grey line represents the consensus threshold set at 70%

**Fig. 4:**
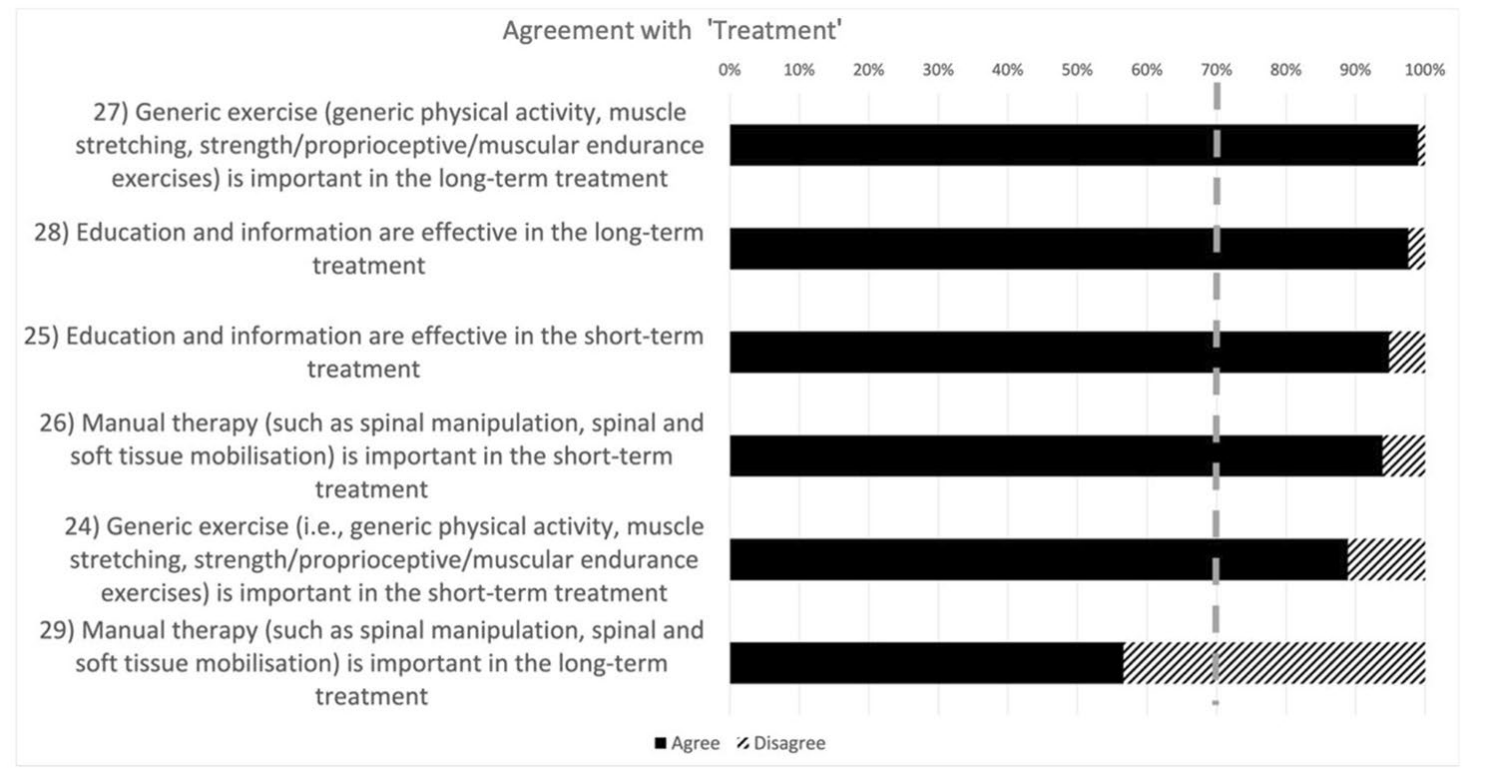
Levels of agreement among physiotherapists on the treatment of non-specific thoracic spine pain. *The dashed grey line represents the consensus threshold set at 70%

Consensus was achieved for 22 (75%) statements (3, 5, 9–28) out of 29 (Figs. 1, 2, 3 and 4). As per the ‘definition’ (Fig. 1), agreement was found in defining non-specific TSP as experienced in the thoracic spine region, between the thoracic levels T1-T12 and across the posterior side of the trunk (statement 1). Most participants agreed that non-specific TSP is experienced in the thoracic region but has a multisystemic origin with several factors (biological, social, neurophysiological, psychological) influencing the onset of non-specific TSP (statement 5). However, no agreement was found with all the other statements regarding the definition of non-specific TSP (statements 1, 2, 4, 6, 7).

When it came to the ‘Assessment’ (Fig. 2), participants agreed with the importance of formulating the clinical diagnosis of non-specific TSP and the role of the clinical interview, physical examination, and manual tests in assessing non-specific TSP (statements 9–17). Patient history was considered a fundamental tool to diagnose non-specific TSP by most participants (> 95%). Conversely, no agreement was found in the statement addressing radiographic findings’ role in non-specific TSP clinical assessment (statement 8).

As far as the ‘Psychosocial factors’ are concerned (Fig. 3), all participants agreed with all the statements dealing with the importance and influence of psychological factors (kinesiophobia, mental health issues, contextual and social factors etc.) in non-specific TSP (statements 18–23). As per the ‘treatment’ (Fig. 4), agreement was found with the statements addressing the role of exercise and education in the short- and long-term treatment of non-specific TSP (statements 24, 25, 27, 28). Conversely, agreement on manual therapy efficacy in non-specific TSP was only reported in the short-term treatment (statement 26) but not in the long one (statement 29).

**Fig. 5:**
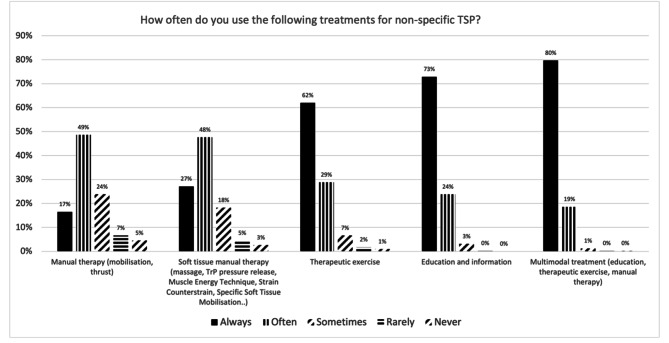
Frequency of treatment techniques to treat non-specific thoracic spine pain

With regards to the treatment approach, most participants (79.7%) indicated they would “always” adopt a multimodal treatment approach (education, therapeutic exercise, manual therapy), followed by education and information (72.9%), therapeutic exercise (62.0%), soft tissue manual therapy (27.1%), and manual therapy (16.5%). Please refer to Fig. 5.

## Discussions

This cross-sectional online survey-based study focussed on physiotherapists’ clinical management of non-specific TSP. As the challenge of managing non-specific TSP persists, understanding current physiotherapy clinical practice for TSP is fundamental to inform future research [3]. Regarding the definition of non-specific TSP, we found different and contrasting results. Most of the participants agreed with the definitions reported by Briggs et al. [4] and the multisystemic origin of non-specific TSP. However, no agreement was found with all the other statements regarding the definition of non-specific TSP. This result reflects the confusion and little clarity in defining non-specific TSP. An accurate definition of the non-specific origin of thoracic spine pain (TSP) is currently absent in the literature, unlike LBP and NP for which a definition exists [31, 32]. Future studies should address this issue.

As far as the clinical assessment is concerned, our findings showed that almost all participants consider the patients’ history essential to formulate a clinical diagnosis of non-specific TSP rather than imaging. Current evidence highlights the importance of exploring patient history thoroughly for identifying contraindications and precautions for treatments, generating a hypothesis or functional diagnosis and physical examination planning [33]. However, as Myburgh reported [34], thoracic spine radiographs remain widely used as a first-line investigative method and are often requested in patients with non-specific TSP. All CPGs for non-specific LBP and NP do not recommend routine imaging [35]. Radiographic findings should be considered only when other diseases are suspected of causing the symptoms (e.g. infection, cancer, rheumatoid arthritis) or when a surgical intervention is planned [36].

Consensus was also achieved for the statements on the importance and influence of psychological factors in non-specific TSP. LBP and NP CPGs recommend considering and assessing psychosocial factors (kinesiophobia, pain catastrophising, contextual, social and work-related factors, and mental health issues) for diagnostic purposes and treatment planning within a biopsychosocial model [36] due to their impact on pain perception [37–42]. The biopsychosocial model considers not only biological but also psychological and social factors as determinants of pain perception [43]. It considers the multidimensionality of pain as determined by different interlaced dimensions (sensory-discriminative, cognitive-evaluative, motivational-affective, behavioural and social) [43]. Clinicians are encouraged to explore these dimensions while assessing an individual’s pain experience and considering them during treatment. ‘Psychologically informed physiotherapy’ interventions (e.g., graded exposure, acceptance and commitment and cognitive behavioural interventions) have been tested on other RMD conditions (e.g., LBP and complex regional pain syndrome) [43, 44]. Therefore, future studies should test their effect on non-specific TSP.

When it came to the treatment, we asked the participants which strategies (manual therapy, education, exercise) they considered helpful in the short and long term. Most participants reported that patient education and physical exercise are essential, and they were the most preferred short-term and long-term strategies. According to LBP[18] and NP [19] CPGs, the most promising approaches seem to involve physical activity and exercise together with appropriate (biopsychosocial) education. On the other hand, most participants did not consider it essential to include manual therapy (e.g. spinal manipulation, spinal mobilisation, soft tissue mobilisation) in the long-term, but only in the short-term treatment. This is in line with current evidence showing that once manual therapy is compared with exercise therapy alone, it provides only short-term benefits in pain reduction, function and physical performance improvement [45]. However, manual therapy is mainly recommended as a component of multimodal care, along with other strategies, including exercise, psychological therapies, information/education, and activity advice, rather than a stand-alone treatment [46]. A multimodal treatment is suggested by NP [19] and LBP [18] CPGs and a recent systematic reviews of CPGs to manage musculoskeletal pain [46]. This approach aligns with the results of this study as the use of a multimodal treatment was proposed by the majority of our participants also for non-specific TSP. However, we only tested the prevalence of the use of these treatments. Future qualitative or mixed-method studies should explore physiotherapists’ TSP clinical decision-making in-depth in the absence of CPGs.

Our study had some limitations that should be acknowledged. Firstly, the findings of our study are based on the results of a survey instrument without response rate calculation. To be noted is that the number of participants indicated by the sample size calculation was achieved. The survey instrument utilised for the study was created ad hoc by the RMD experts for the study and validity and reliability of the survey instrument was not calculated. It could be hypothesised that a degree of sampling bias was introduced in the study as physiotherapists interested in non-specific TSP would have been more willing to participate in this study. Then, most participants were recruited through an invitation via the newsletter of a postgraduate master’s degree in RMD rehabilitation, limiting the generalisability of the findings to those without such educational attainment. The main strength of this study is that it is the first study to focus on physiotherapists’ management of non-specific TSP, as other studies mainly concentrated on LBP and NP. Moreover, it is the first study that investigated a population from a Mediterranean area. Health professionals from southern countries reported higher educational needs than their northern counterparts [47]. Hence, retrieving information from these populations is necessary to promote education campaigns to bridge the gap between European countries. Having said this, the statement can also be considered a further limitation as our findings cannot be generalised to other countries.

Despite the lack of evidence, the treatment options identified by the participants of this study are consistent with the treatments proposed in NP and LBP CPGs. This brings to the forefront promising results about the professional development of physiotherapists in Italy in the field of RMD rehabilitation. However, some grey areas are yet to be addressed in the management of non-specific TSP. Firstly, the development of non-specific TSP CPGs is crucial. Secondly, it is pivotal to promote the use of these guidelines in university programmes. Lastly, facilitating the timely translation of evidence for non-specific TSP into clinical practice will be another step to deeply impact on non-specific TSP quality of care.

## Electronic supplementary material

Below is the link to the electronic supplementary material.

Supplementary Material 1

## Data Availability

The datasets used and analysed during the current study are available from the corresponding author upon reasonable request.
