# Supplementary material for: Management of non-specific thoracic spine pain: a cross-sectional study among physiotherapists
Source: BMC Musculoskelet Disord. 2023 May 19;24:398. doi: 10.1186/s12891-023-06505-8 (PMC10197218; doi:10.1186/s12891-023-06505-8)
Supplement: Supplementary file 1 — Supplementary Material 1 [file 12891_2023_6505_MOESM1_ESM.docx]

**Supplementary file 1**

**MANAGEMENT OF NON-SPECIFIC MUSCULOSKELETAL THORACIC PAIN: A CROSS-SECTIONAL STUDY AMONG ITALIAN PHYSIOTHERAPISTS**

**Section 1 - Demographic, work and academic characteristics of the sample**

1. How old are you?

- 20-30
- 31-40
- 41-50
- 51-60
- >60

1. Gender you identify with:

- Man
- Woman
- Others (please state)

1. Years of practice as physiotherapist:

- < 5
- 6-10
- 11-20
- > 20

1. Region in which you attended your Bachelor of Science in Physiotherapy:

- Abruzzo
- Aosta Valley
- Apulia
- Basilicata
- Calabria
- Emilia-Romagna
- Friuli-Venezia-Giulia
- Lazio
- Liguria
- Lombardy
- Marche
- Molise
- Piedmont
- Sardinia
- Sicily
- Trentino-South Tyrol
- Tuscany
- Umbria
- Veneto
- Foreign country

1. Name of the University where you got you Bachelor Degree

_________________________________________

1. In which region do you work?

- Abruzzo
- Aosta Valley
- Apulia
- Basilicata
- Calabria
- Emilia-Romagna
- Friuli-Venezia-Giulia
- Lazio
- Liguria
- Lombardy
- Marche
- Molise
- Piedmont
- Sardinia
- Sicily
- Trentino-South Tyrol
- Tuscany
- Umbria
- Veneto
- Foreign country

1. What is your highest academic level reached?

- Post-Graduate I Level Degree
- Master of Science (MSc)/Post-Graduate II Level Degree
- Doctor of Philosophy (PhD)
- Other
- Nothing

1. What is your main health sector?

- Employee in public sector
- Employee in private sector
- Freelancer
- Coordinator
- Hospital
- Manager

1. What is your predominant physiotherapy practice specialty?

- Rheumatic and Musculoskeletal Diseases/orthopaedics
- Neurology
- Cardiorespiratory
- Geriatric
- Paediatric
- Pelvic floor
- Sport/Performing Arts
- Hand rehabilitation
- Management
- Research
- University

1. Considering your clinical expertise, how much people with non-specific TSP do you treat per year?

- <10
- 10-20
- >20

1. In the first session, do you investigate psychosocial factors in patients with non-specific TSP?

- Yes
- No

**DEFINITION**

**Please indicate your agreement with the following sentence using a 5-point Likert scale ranging from completely disagree (score 1) to completely agree (score 5)**

| 1. How much do you agree with the definition of “Non-specific TSP”? | | | | | |
| --- | --- | --- | --- | --- | --- |
|  | **Completely disagree** | **Disagree** | **Neither agree nor disagree** | **Agree** | **Completely agree** |
| Non-specific TSP is experienced between the thoracic levels T1-T12 and the most lateral margins of the erector spinae muscles |  |  |  |  |  |
| Non-specific TSP is experienced between the thoracic levels C7-T1 and T12-L1, centrally to the spine |  |  |  |  |  |
| Non-specific TSP is experienced in the region of the thoracic spine, between the thoracic levels T1–T12 and across the posterior side of the trunk |  |  |  |  |  |
| 1. In your experience, how much do you agree with the following sentences regarding the concept of non-specificity in TSP? | | | | | |
|  | **Completely disagree** | **Disagree** | **Neither agree nor disagree** | **Agree** | **Completely agree** |
| It is not possible to identify clinically, via palpation and / or through instrumental tests a specific musculoskeletal structure as the source of back pain |  |  |  |  |  |
| Non-specific TSP is experienced in thoracic region but has multisystemic origin |  |  |  |  |  |
| It is not necessary to identify a specific musculoskeletal structure that can be defined as the source of non-specific TSP |  |  |  |  |  |
| It is possible to identify to identify clinically, via palpation and / or through instrumental tests a specific musculoskeletal structure that can be defined as the source of back pain. |  |  |  |  |  |

**ASSESSMENT**

**Please indicate your agreement with the following sentence using a 5-point Likert scale ranging from completely disagree (score 1) to completely agree (score 5)**

| 1. In your experience, how much do you agree that the following elements are useful in diagnosing the non-specific TSP clinically? | | | | | |
| --- | --- | --- | --- | --- | --- |
|  | **Completely disagree** | **Disagree** | **Neither agree nor disagree** | **Agree** | **Completely agree** |
| Chest imaging (MRI, CT, RX) |  |  |  |  |  |
| Clinical Interview |  |  |  |  |  |
| Manual tests |  |  |  |  |  |
| Physical examination |  |  |  |  |  |
| 1. How much do you agree with the following assessment techniques in diagnosing the non-specific TSP? | | | | | |
|  | **Completely disagree** | **Disagree** | **Neither agree nor disagree** | **Agree** | **Completely agree** |
| Active movements observation |  |  |  |  |  |
| Neurological examination |  |  |  |  |  |
| Passive range-of-motion examination |  |  |  |  |  |
| Soft tissue palpation |  |  |  |  |  |
| Regional and segmental joint provocation tests |  |  |  |  |  |
| Regional and segmental passive tests |  |  |  |  |  |

**PSYCHOLOGICAL FACTORS**

**Please indicate your agreement with the following sentence using a 5-point Likert scale ranging from completely disagree (score 1) to completely agree (score 5)**

| 1. In your experience, how much do you agree with the importance of psychosocial factors in investigating non-specific TSP? | | | | | |
| --- | --- | --- | --- | --- | --- |
|  | **Completely disagree** | **Disagree** | **Neither agree nor disagree** | **Agree** | **Completely agree** |
| 1. In your experience, how much do you agree with the following factors influencing pain in the patient with non-specific TSP? | | | | | |
|  | **Completely disagree** | **Disagree** | **Neither agree nor disagree** | **Agree** | **Completely agree** |
| Kinesiophobia |  |  |  |  |  |
| Pain catastrophising |  |  |  |  |  |
| Mental disorders (e.g., anxiety disorders and major depression) |  |  |  |  |  |
| Contextual and social factors |  |  |  |  |  |
| Social factors work-related |  |  |  |  |  |

**MANAGEMENT AND TREATMENT**

**Please indicate your agreement with the following sentence using a 5-point Likert scale ranging from completely disagree (score 1) to completely agree (score 5)**

| 1. In your experience, how much do you agree with the importance in using the following types of treatment in the short term? | | | | | |
| --- | --- | --- | --- | --- | --- |
|  | **Completely disagree** | **Disagree** | **Neither agree nor disagree** | **Agree** | **Completely agree** |
| Generic exercise (i.e., generic physical activity, muscle stretching, strength/proprioceptive/muscular endurance exercises) |  |  |  |  |  |
| Education and information |  |  |  |  |  |
| Manual therapy (such as spinal manipulation, spinal and soft tissue mobilisation) |  |  |  |  |  |
| 1. In your experience, how much do you agree with the importance in using the following types of treatment in the long term? | | | | | |
|  | **Completely disagree** | **Disagree** | **Neither agree nor disagree** | **Agree** | **Completely agree** |
| Generic exercise (i.e., generic physical activity, muscle stretching, strength/proprioceptive/muscular endurance exercises) |  |  |  |  |  |
| Education and information |  |  |  |  |  |
| Manual therapy (such as spinal manipulation, spinal and soft tissue mobilisation) |  |  |  |  |  |

**Please indicate your agreement with this sentence using a 5-point scale (always – often – sometimes – rarely – never)**

| 1. In your experience, how often do you use the following types of treatment for non-specific TSP? | | | | | |
| --- | --- | --- | --- | --- | --- |
|  | **Always** | **Often** | **Sometimes** | **Rarely** | **Never** |
| Manual therapy (mobilisation, thrust) |  |  |  |  |  |
| Soft tissue manual therapy (massage, TrP pressure release, Muscle Energy Technique, Strain Counterstrain, Specific Soft Tissue Mobilisation..) |  |  |  |  |  |
| Therapeutic exercise |  |  |  |  |  |
| Education and information |  |  |  |  |  |
| Multimodal treatment (education, therapeutic exercise, manual therapy) |  |  |  |  |  |
| Manual therapy (such as spinal manipulation, spinal and soft tissue mobilisation) |  |  |  |  |  |
